# A new *Xenicocephalus* species from Ecuador (Heteroptera, Enicocephalomorpha, Enicocephalidae)

**DOI:** 10.3897/zookeys.796.24538

**Published:** 2018-11-15

**Authors:** Pavel Štys, Petr Baňař

**Affiliations:** 1 Charles University, Faculty of Science, Department of Zoology, Viničná 7, CZ-128 44 Praha 2, Czech Republic Charles University Prague Czech Republic; 2 Moravian Museum, Department of Entomology, Hviezdoslavova 29a, Brno, CZ-627 00, Czech Republic Moravian Museum Brno Czech Republic

**Keywords:** Abdomen, biology, differential diagnoses, distribution, Ecuador, forelegs, Hemiptera, new species

## Abstract

*Xenicocephalustomhenryi***sp. n.** (Insecta: Hemiptera: Heteroptera: Enicocephalomorpha: Enicocephalidae) is established for a single macropterous female from Ecuador. The enigmatic genus now includes three species known from only two Neotropical adults and an incomplete female specimen. The new species is described and illustrated, extensive comparative diagnoses for *Xenicocephalus* species are provided, and nomenclature, distribution, and biology of the genus are reviewed. The architecture of the raptorial forelegs of *Xenicocephalus* is unique among Enicocephalomorpha, and the genus is classified as subfamily *incertae sedis*.

## Introduction

A new Neotropical genus and species of Enicocephalidae (Enicocephalinae), *Xenicocephalusgiganticus* Wygodzinsky & Schmidt, 1991, was well described and illustrated by Wygodzinsky and Schmidt (1991) from a single incomplete female specimen from Colombia. Wygodzinsky and Schmidt (1991) also provided miscellaneous notes on the taxon and described the larvae, and we liberally use data from their paper. Štys (2002) included *Xenicocephalus* in a global key to genus-group taxa of the infraorder Enicocephalomorpha. Štys and Baňař (2008a) described a second species of the genus, *Xenicocephalusjosifovi* Štys & Baňař, from Suriname based on a single male, discussed various aspects of morphology and provided a new diagnosis of the genus. Nothing else has been written on this strange taxon, and only one and a half adult specimens are known. Wygodzinsky’s prediction “I am hopeful that adult material will soon become available and that a more complete description can be made”(Wygodzinsky and Schmidt 1991) is marginally met by our discovery of a single female specimen of a new species from Ecuador, which is described and discussed herein.

Owing to the specimen’s uniqueness, we could not study its anatomy in depth. Our descriptions of the head, antennae, pronotum and forelegs in an adult female, however, are the first for the genus. The female holotype of *X.giganticus* lacked these body parts, and the genus description had to be supplemented (Wygodzinsky and Schmidt 1991) by data from the last instar larva (probably conspecific and a female), which best approximates the female condition.

In the Discussion we emphasize some systematic issues concerning *Xenicocephalus* that have never been considered, comprehensively review certain aspects of biology, and briefly mention the construction of the fore leg of *Xenicocephalus*, which is unique among Enicocephalomorpha. Their architecture will be considered in a separate paper assessing the suprageneric phylogenetic classification of the Enicocephalomoropha (Štys and Baňař, in prep.).

## Materials and methods

The term ocular index refers to the ratio of the minimum interocular distance to the maximum width of the eye; it is best calculated if measured as twice minimum interocular distance/maximum width across the eyes, minus minimum interocular distance. Measurements were taken using a SZP 11 ZOOM stereoscopic microscope with an eyepiece micrometer.

Color photographs of the newly described species were taken with a Leica MSV266 camera. Scanning electron micrographs of a gold-coated left foreleg were taken using a JEOL 6380 LV scanning electron microscope.

Label data are cited verbatim, including potential errors, using a slash (/) to separate lines on the label; different labels are mentioned and indicated by a double slash (//). Our notes are in [square brackets].

For simplicity, our nomenclature for veins and cells follows that used by Štys (2002: Figure 1).

**Figure 1.**
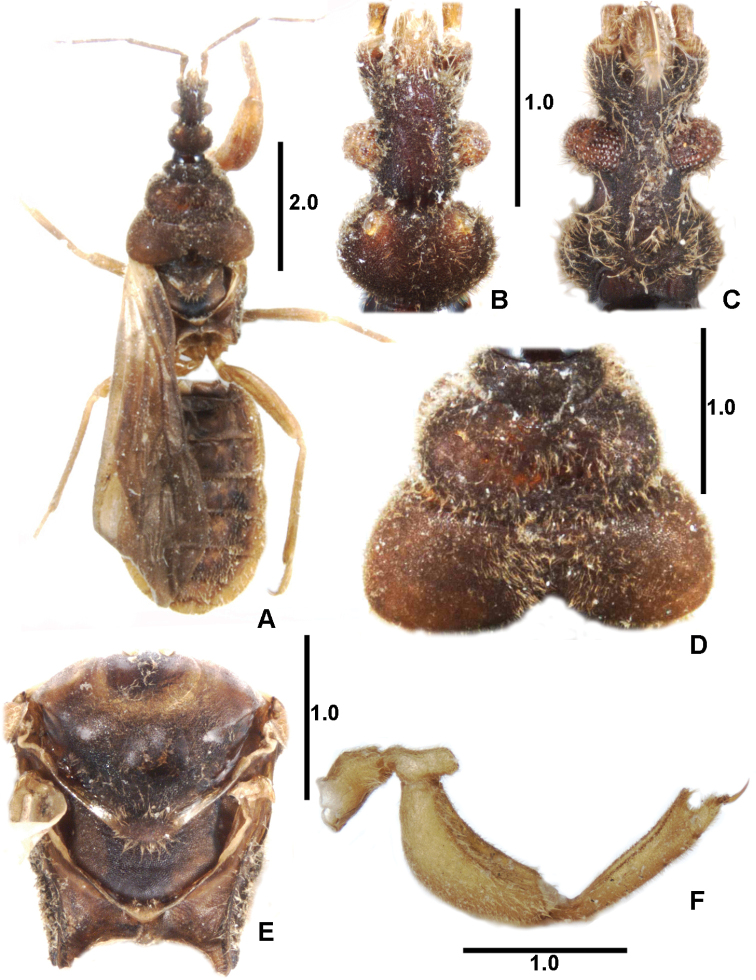
*Xenicocephalustomhenryi* sp. n., female holotype **A** dorsal habitus **B** head, dorsal view **C** head, ventral view **D** pronotum, dorsal view **E** pterothorax, dorsal view **F** left foreleg, posterior view. Scale bars in mm.

Abbreviations used in the text:

**DLTG** dorsal laterotergite;

**F** female;

**L** larva of the fifth instar;

**M** male;

**MTG** mediotergite.

## Taxonomy

### Infraorder Enicocephalomorpha Stichel, 1955

#### Family Enicocephalidae Stål, 1860

##### Genus Xenicocephalus

Taxon classificationAnimaliaHemipteraEnicocephalidae

Wygodzinsky & Schmidt, 1991

###### Type species.

*Xenicocephalusgiganticus* Wygodzinsky & Schmidt, 1991 by original designation.

##### Xenicocephalus tomhenryi sp. n.

Taxon classificationAnimaliaHemipteraEnicocephalidae

http://zoobank.org/A16BB301-C41B-44CB-8426-B04E2C8C625F

**Figure 2.**
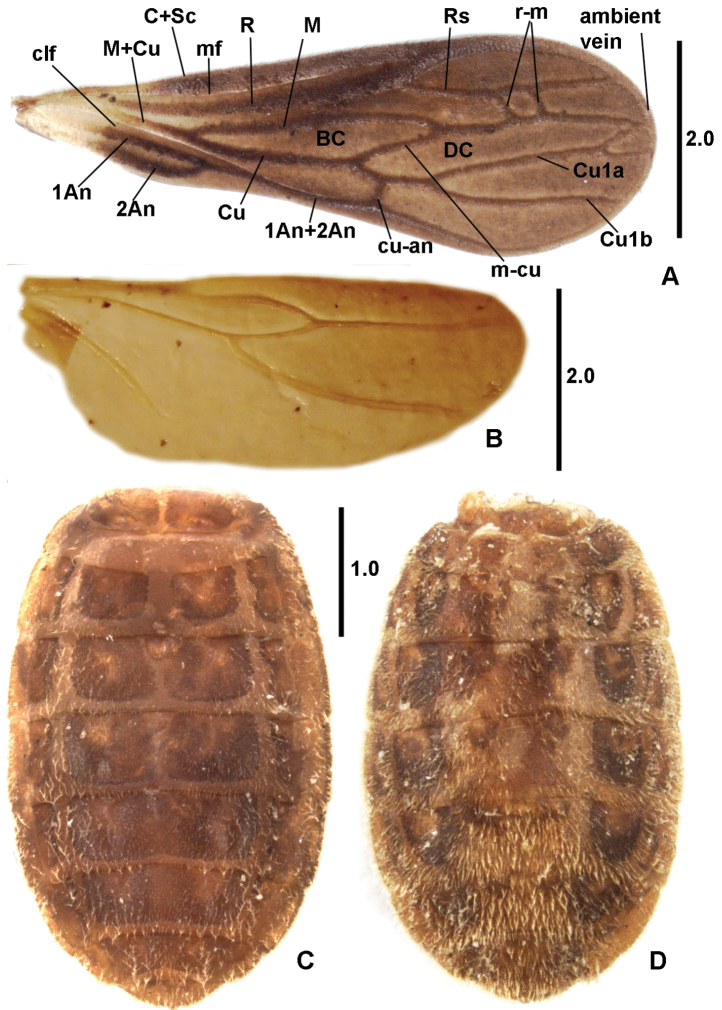
*Xenicocephalustomhenryi* sp. n., female holotype **A** forewing **B** hindwing **C** abdomen, dorsal view **D** abdomen, ventral view. Scale bars in mm.

**Figure 3.**
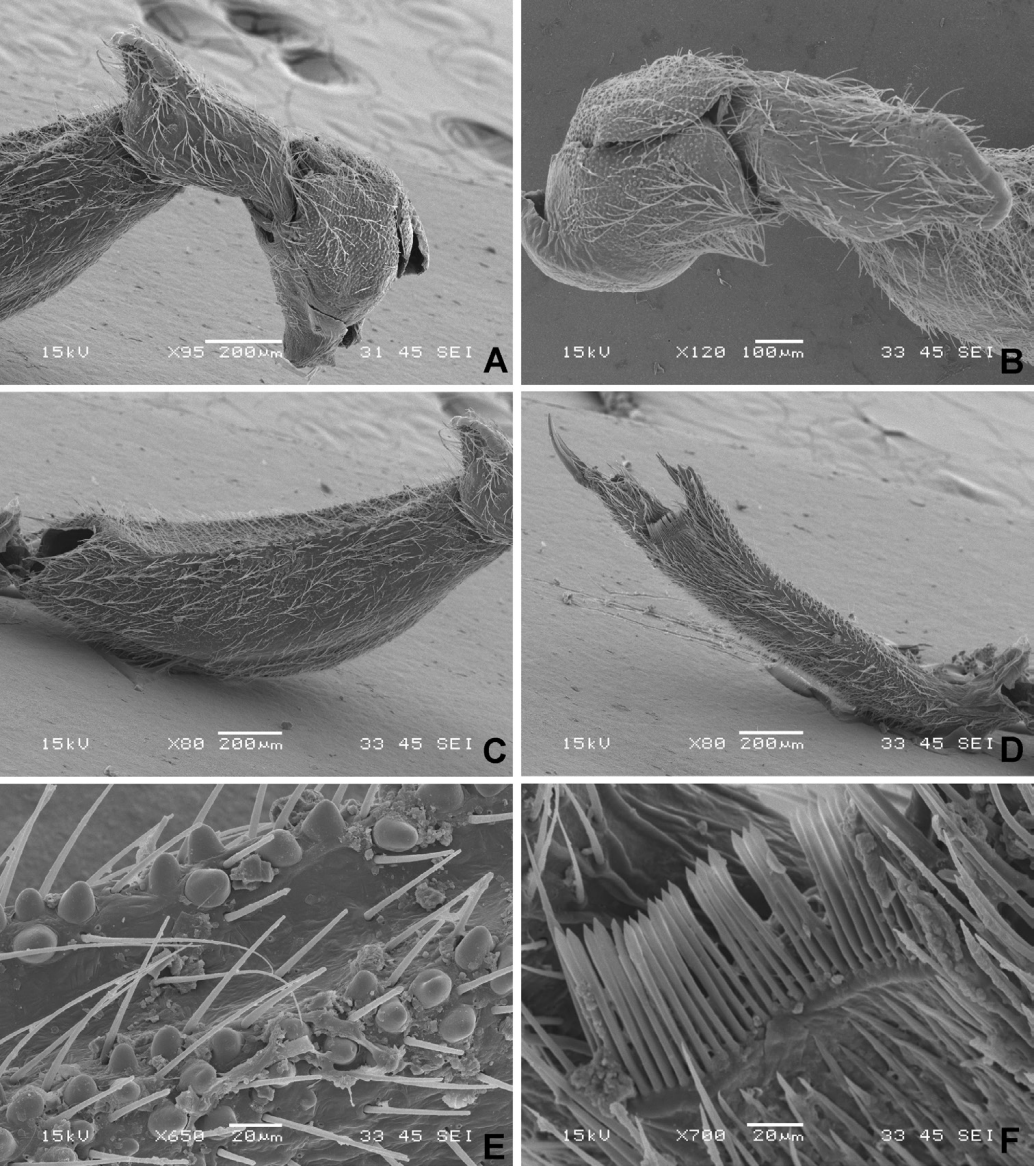
*Xenicocephalustomhenryi* sp. n., female holotype, left foreleg, scanning electron micrographs **A** coxa, trochanter and basis of femur, anterior view **B** coxa and trochanter, ventral view **C** femur, anterior view **D** tibia, anterior view **E** detail of ventral concavity of tibia **F** bristle comb of tibial apex, anterior view.

**Figure 4.**
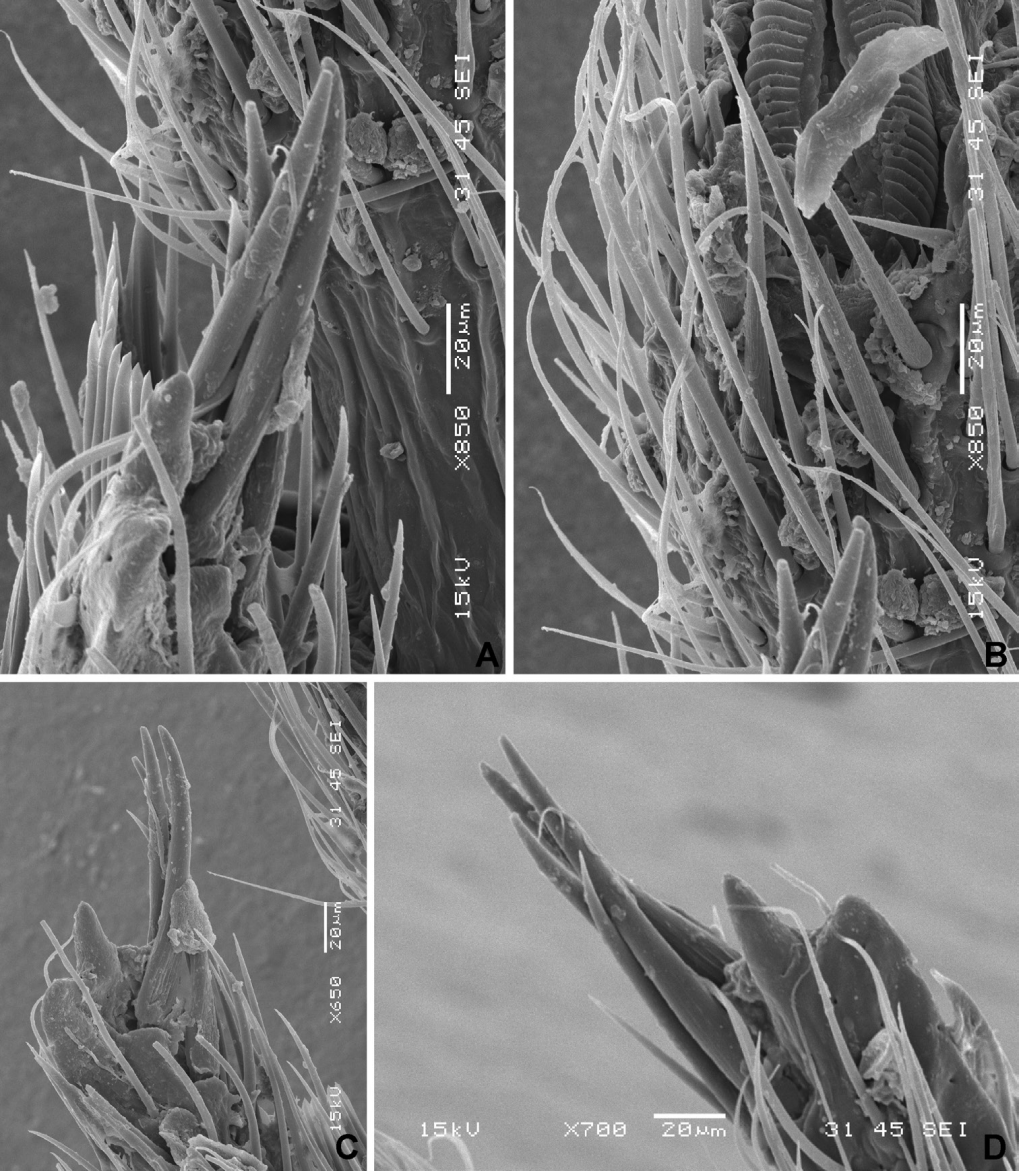
*Xenicocephalustomhenryi* sp. n., female holotype, left foreleg, scanning electron micrographs **A** apicitibial armature, ventral view **B** tarsal armature, ventral view **C** apicitibial armature, posterior view **D** apicitibial armature, anterior view.

**Figure 5.**
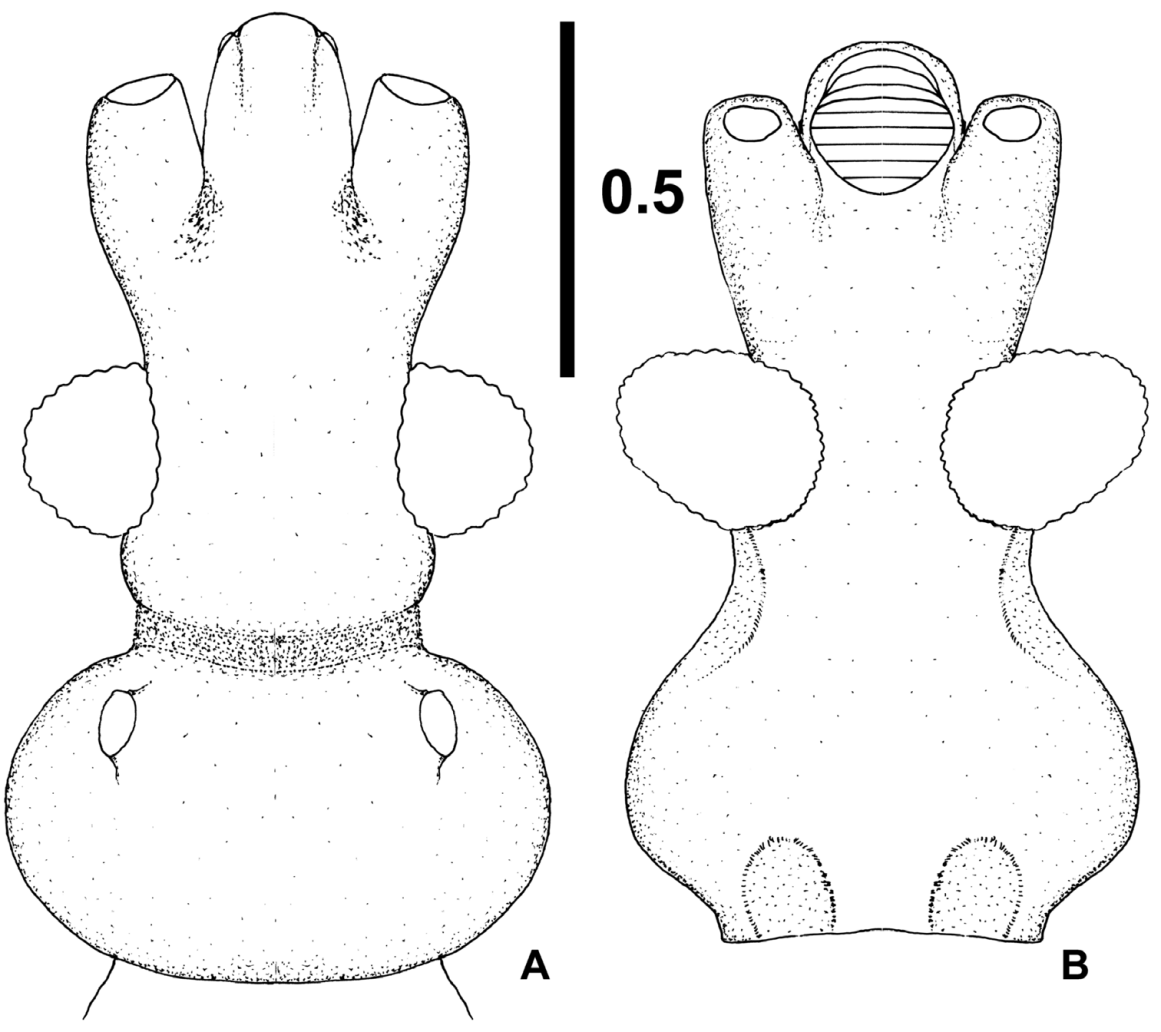
*Xenicocephalustomhenryi* sp. n., female holotype, head **A** dorsal view **B** ventral view. Scale bar in mm.

**Figure 6.**
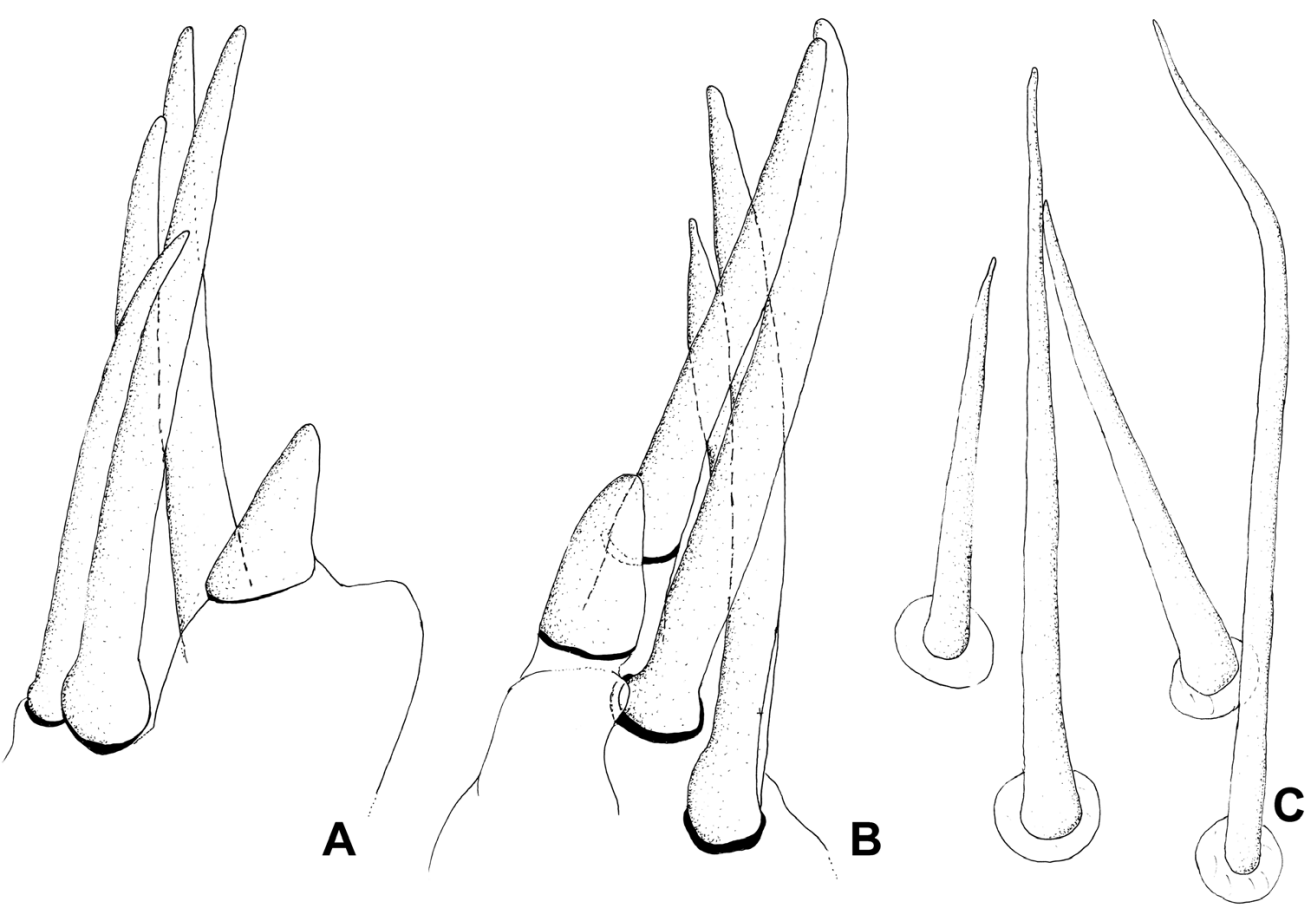
*Xenicocephalustomhenryi* sp. n., female holotype, left foreleg, schemes, not measured. **A** apicitibial armature, antero-ventral view **B** apicitibial armature, ventral view **C** tarsal armature, ventral view, other macrotrichia omitted.

Figures 1 , 2 , 3 , 4 , 5 , 6

###### Description.

Measurements (in mm) of female holotype; L = length; W = width. Total body L 8.60. Head: total L (without neck) 1.31; posterior lobe, L 0.51, posterior lobe, W 0.71; distance eye-apex of antennifer 0.37; maximum width across eyes 0.67; dorsal minimum interocular distance 0.33; ventral minimum interocular distance 0.20; eye L 0.22. Labium total L 0.60. Antenna: Segment I L 0.40; segment II L 0.96, segment III L 0.89, segment IV L 0.82. Pronotum: total L (maximum) 1.64; collum, L (median) 0.28, maximum W 0.78; midlobe, L (median) 0.64, midlobe, W (maximum) 1.42; hindlobe, L (maximum) 0.87, hindlobe, L (median) 0.40; hindlobe, W (maximum) 2.02. Foreleg: femur L 1.49, femur, maximum W 0.51, tibia L 1.36, tibia maximum W 0.29. Forewing L 5.95, W 2.18. Hindwing L 4.94, W1.92.

***Coloration*** dark brown (Figure 1A), legs, labium, antennomere IV and base of forewing paler, light brown.

***Pilosity*.** Antennae densely covered with short, semi-erect setae, dorsal and lateral parts of head covered with long, semi-erect setae of variable orientation, mixed with shorter, erect setae similar to those on antennae and compound eyes. Venter of head with long semi-erect setae. Vestiture on pronotum, lateral, and ventral parts of thorax similar to that on dorsum of head. Forelegs with numerous long semi-erect setae of different orientation, setae on ventral face of foretibia shorter and erect. Forewing veins with short semi-erect to erect setae; hindwing veins bare. Dorsum of abdomen with semi-erect and erect setae, longest on “outer” laterotergites, becoming shorter to nearly absent toward medial parts of mediotergites. Lateral faces of laterotergites only with short, erect setae. Venter of abdomen densely covered with vestiture, becoming denser and longer towards apex of abdomen.

***Texture*.** Body faces, including forewing, densely covered with countless cuticular microgranules, giving a matt appearance. Foretibia along almost entire ventral face (except proximal sixth) with two rows (posterior and anterior one) of irregularly placed large, semi-globular to slightly conical, strongly sclerotized granules (Figure 3D, E), appearing under lower magnification as impression of two deeply brown to blackish lines (Figure 1F). Forefemur similar but with anterior row of large granules, more developed, situated on slightly elevated rim; granules in posterior row smaller, nearly subglobular proximally, becoming lens-like platelets distally. Surface of forefemoral concavity with countless lens-like platelets.

###### Structure.

***Head*.** Rather narrow, strongly elongate (Figs 1B, C, 5A, B). In dorsal view, preocular margins (formed largely by antennifers) strongly diverging distad, moderately convex, much shorter than strongly produced medial clypeo-mandibular projection. Eyes prominent, semiglobular, with numerous separate, convex facets, eye width much shorter than synthlipsis, dorsal ocular index 1.94. Postocular part and postocular constriction (long and shallow) hardly distinguishable from each other, long, laterally strongly concave, about as long as antero-posterior length of eye. Postocular lobe short and broad, widest in middle, slightly wider than transocular width, lateral sides broadly rounded. Ocellar tubercles strikingly large and prominent, large ocelli directed anterolaterad: ocellar tubercles in Enicocephalomorpha bear ocelli dorsally or dorsolaterally; entire dorsal surface of ocellar tubercles sometimes occupied by ocelli with their tubercles hardly indicated. Tubercles and ocelli are mostly not distinguished in descriptive papers (including some of ours). Interocellar distance 1.5 times as long as distance of ocellar tubercle to eye. Neck simple. Eyes in ventral view strongly produced laterad and sunken mesad, longest axis strongly diagonal and directed antero-laterad, eye much wider than minimum interocular distance, ventral ocular index 0.85.

***Antennae*.** Insertion nearly subterminal, segment 1 with a large prescapite, segment itself strongly widening distad, much surpassing apex of head, segment 2 terete, slightly thickening distad, segments 3 and 4 long, thinner, but less than subflagelliform. Antennal formula (longest segment first): II:III:IV:I.

***Labium*.** Short, reaching anterior margins of eyes. Labial formula (longest segment first) III-IV-II=I. The dorsal (morphologically ventral) outline of segment III moderately convex, ventral one straight.

***Pronotum*** (Figure 1D). Lateral and posterior outlines of collum large and moderately sized, wider midlobe strongly convex, midlobe laterally much exceeded by moderately convex lateral margins of hindlobe. Convex posterior margin of hindlobe subdivided into two separately convex parts by deep subtriangular median gulf reaching nearly half length of hindlobe median. Details of surface shape of pronotal lobes undetectable. Ratio width to median length of collum 2.78; of the midlobe 2.22 and ratio of maximum width of hindlobe to maximum length 2.32.

###### Other parts of thorax.

Fore acetabula widely separated and widely open. Mesoscutellum broadly triangular, apex not produced but provided with transversely oval swelling with marginally radiating macrotrichia. Mesonotum and metanotum (Figure 1E).

***Forelegs*.** Foretrochanter (Figs 1F, 3A, B) produced as subtriangular process over anterior base of forefemur, anterior margins of latter diagonal and thickened by marginal inversely L-shaped ridge; posterior margins of process perpendicular to femur, posterior face of process concave. Only areas ventral to process and small inner angles of inversely L-shaped ridges with granules, remainder of trochanter smooth.

Forefemur (Figs 1F, 3C) strikingly thick and curved, arcuate, dorsal face convex, ventral face deeply and percurrently concave; groove delimited by two marginal rows of densely packed minute subglobular platelets, surface of concavity densely pilose. (Neopatella [cf. Štys and Baňař 2007] not studied.)

Foretibia (Figs 1F, 3D) thick, flattish, rather uniformly broad, very moderately arcuate, ventral face deeply percurrently concave and densely pilose, concavity about as long as that of forefemur, margins of concavity delimited as in forefemur by subglobular platelets (Figure 3E). Apicitibial armature (Figs 4A, C, D, 6A, B). Tibial process long and thin, about as long as foretarsus, strikingly delimited from remainder of tibia by concavity containing medial part of bristle-comb formed by ca. 28 palisade-like spines (Figure 4B). Apiciventral tibial spines formed by two groups: (a) more dorsal cluster of four long, thin, slightly curved spines (their proximal parts with parallel grooves), cluster separated from (b) more ventral area by shorter, thicker, and less pointed, finger-shaped spine above conspicuous ventral-most rounded projection of process. Foretarsus. Tarsus with four (Figs 4B, 6C) somewhat thicker setae (two anterior + two posterior = two dorsal + two ventral) distributed among normal macrotrichia, not longer than these, and recognizable only by presence of longitudinal grooving.

***Midlegs and hindlegs*** short and robust, first tarsomeres very short. Apices of middle and hind tibiae with two bristle combs, anterior and posterior ones each consisting of ca. 12–15 isomorphic setae, and two or three much longer, ventrally placed setae.

***Post-tarsi*** could not be studied in detail. Fore post-tarsus with strikingly split unguitractor plate along median. Claws heteromorphic, strikingly slender and short, longest at foreleg (posterior claw reduced to stump), shortest at midleg. Fore claw and hind claws nearly straight, middle claw basally thick and distally regularly arcuate.

***Forewing*** (Figure 2A) veins with short, semi-erect to erect setae. Basal and discal cells closed; indistinct ambient vein marginal, coinciding with wing margin; costal fracture (or node) absent. Veins C+Sc and R and R1 so strongly thickened that anteradial furrow displaced laterad at inner edge of C&Sc. Medial furrow (= postradial furrow) absent. Basal anchor-like vein short and thick; distinctly diagonal, vein-like r-m entering MA in middle of discal cell (r-m teratologically doubled on right wing). M and Cu branching rather proximally; basal cell thus long and pointed but still slightly shorter than pointed discal cell. Discal cell nearly reaching wing margin, connecting with it by very short M&Cu1a and longer Cu1b; cu-an entering discal cell much more proximally relative to branching point of Cu1a and Cu1b. AA1+2 and AA3+4 meeting to form joint distal sector of AA in ca. 2/5 length of AA before joining cu-an. Entire wing diffusely melanized except base.

***Hindwing*** (Figure 2B). ***Pregenital abdomen*** moderately broad, oval, apex nearly pointed. ***Dorsum*** with peculiar system of elevated longitudinal (lateral and medial) rails and transverse rungs resembling lattice of ladder (Figure 2C). MTG I subdivided into two hemitergites by median desclerotization; separate LTG lacking. MTG II very short, strongly thickened, forming first rung of “ladder”; LTG I & II laterally embracing MTG I. Mediotergites III–VIII each with rung along posterior margin (extending on segments III–VI to connexival margin). Segments III–VII with strong lateral rails forming lateral margins of MTG, separating them from DLTG area subdivided in inner DLTG (= lateral-most parts of MTG?) and outer DLTG; subdivision of DLTG area indistinct on dorsite VIII. MTG III–V with distinct and wide (VI with indistinct and diffusing) median rail. Dorsites VIII and IX simple. Opening of dorsal gland at anterior margin of MTG IV formed by semicircular depression provided with pair (sic!) of large, posterior, nearly contiguous openings. Venter (Figure 2D). Details of ventrite I not discernible. Ventrites II–VII with more complex lattice than on dorsum but details not studied. Transverse, narrow, and long genital slit between ventrites VII and VIII; any other genital or proctigeral structures not observed.

###### Type material.

***Holotype*** female, labelled: 'Ecuador camino / Aloag-Tandapi / Pr. Pichincha 2600m / 12.II.1983 A. Roig [handwritten] // Drake Colln. ex / J. Maldonado C. / Coll. 1996 [printed] // Xenicocephalus / sp. nov. / P. Baňař det. 2014 [handwritten, partly printed] // HOLOTYPE / *Xenicocephalus* / *tomhenryi* sp. nov. / P. Štys & P. Baňař det. 2018 [printed red label]’. Dry-mounted, left foreleg and right wings mounted separately; right middletarsus missing. Deposited in Department of Entomology of National Museum of Natural History, Washington, D.C. (USNM).

###### Etymology and dedication.

Dedicated to our dear colleague Thomas J. Henry, eminent student of the Heteroptera, for long-standing cooperation and friendship. Pavel will always remember Tom´s and Katy´s hospitality and kind assistance during his stay at their house in Silver Spring, Maryland, after his mishap in 2014.

###### Species comparison.

The following comparative paragraphs are intended to serve as a diagnosis and comparative diagnosis. Because of the paucity of material, we could not always determine whether the differences are species-specific, sex-specific, or represent individual variation. The last alternative may particularly involve characters of the forewing venation, which is notoriously variable and subject to teratological mutations (cf. Štys 1980, Wygodzinsky and Schmidt 1991, Štys and Baňař 2008b).

The data on *Xenicocephalus* species are organized as follows: (1) *X.tomhenryi* female from Ecuador (holotype), (2) *X.josifovi* male from Suriname (holotype), (3) *X.giganticus* female from Colombia (incomplete holotype), (4) *X.* sp., larva 5 from Colombia: (Santa Marta: San Sebastian de Marago) assumed by Wygodzinsky and Schmidt (1991) to be conspecific with *X.giganticus* and used by them in completing the diagnosis of *Xenicocephalus*. The important autapomorphic diagnostic character states are in boldface type.

***Antennae***

*(1) X.tomhenryi* F – **segment I long, strikingly thickening distad**; II long, terete, slightly thickening distad, about as long as head.

*(2) X.josifovi* M – segment I short, thick, not thickening distad; II short, thicker but not thickening distad, about 1.5 times as long as head.

*(3) X.giganticus* F – ?

*(4) X.giganticus* (?) L 5 – segment I short, thickening distad; II long, terete, not thickening distad, about as long as head.

***Head*** (size of eyes sexually dimorphic?)

*(1) X.tomhenryi* F – preocular part of head long, eyes short, distances anterior margin of eye-insertion of antenna and posterior margin of eye-ocellus longer than maximum length of eye; **postocular part of anterior lobe & transverse constriction strikingly long**; ocelli submarginal.

*(2) X.josifovi* M – preocular part of head short, eyes long, distance anterior margin of eye-insertion of antenna and posterior margin of eye-ocellus much shorter than maximum length of eye; **postocular part of anterior lobe not present, constriction narrow**; ocelli marginal.

*(3) X.giganticus* F – ?

*(4) X.giganticus* (F?) L 5 – preocular part of head long, eyes minute, lateral, distance anterior margin of eye-insertion of antennae as long as maximum length of eye; **postocular part of anterior lobe nearly as long as eye**; constriction deep and narrow; ocelli not mentioned in original description, but their rudiments indicated (Wygodzinsky and Schmidt 1991: fig. 149B, p. 206).

***Pronotum***

*(1) X.tomhenryi* F – **posterior margin of collum strongly convex, encroaching onto midlobe region**; hind lobe “entire,“ median part not differentiated; **anterolateral parts of hindlobe embracing posterolateral parts of midlobe**, posteromedial notch of hind lobe ca. twice as deep as maximum median length of hindlobe.

(2) *X.josifovi* M – posterior margin of collum transverse; **hindlobe “bipartite,” creating impression of two opposite leaves attached to broad and weakly sclerotized median region**; anterolateral parts of hindlobe not extending cephalad, posteromedial notch of hind lobe ca. half as deep as maximum median length of hindlobe.

*(3) X.giganticus* F – ?

*(4) X.giganticus* (?) L 5 – posterior margin of collum very moderately convex; mid- and hindlobes not differentiated.

***Mesoscutellum***

*(1) X.tomhenryi* F – broadly rounded, **apex with transversely oval swelling with radiating marginal macrotrichia.**

*(2) X.josifovi* M – amply triangular, apically mucronate.

*(3) X.giganticus* F – amply triangular, apically mucronate.

*(4) X.giganticus* (?) L 5 – 0.

***Forewings*** (individual variation and most potential teratologies could not be assessed). We are not certain about the presence of AP in any *Xenicocephalus* species (contrary to our previous statement on *X.josifovi* (Štys and Baňař 2008a).

*(1) X.tomhenryi* F – C&Sc, R and Rs extremely strongly thickened, **anteradial furrow along edge of C&Sc**; veins delimiting base of discal cell (part of M and part of Cu proximal to cu-an) unequally long, M about three times as long as Cu; **r-m vein-like**; **apex of discal cell close to wing margin, 2 short distal veins entering wing margin**; fork Cu1a-Cu1b far distad to cu-an. Forewing macropterous, conspicuously exceeding apex of abdomen.

*(2) X.josifovi* M – C&Sc, R and Rs moderately thickened, anteradial furrow within subcostal cell; veins delimiting base of discal cell (part of M and part of Cu proximal to cu-an) equally long; **r-m point-like**; apex of discal cell close to wing margin,1 hardly distinct distal vein entering wing margin; fork Cu1a-Cu1b coinciding with position of cu-an. Forewing macropterous, exceeding apex of abdomen.

*(3) X.giganticus* F – C&Sc, R and Rs extremely strongly thickened but anteradial furrow within subcostal cell; veins delimiting base of discal cell (part of M and part of Cu proximal to cu-an) equally long; **r-m vein-like**; apex of discal cell distant from wing margin,1 distinct vein reaching wing margin; fork Cu1a-Cu1b far distad to cu-an. Forewing submacropterous, not exceeding apex of abdomen.

*(4) X.giganticus* (?) L 5 – 0.

***Foretrochanter*** (perceived shape strikingly dependent on angle of observation).

*(1) X.tomhenryi* F – **broadly rounded, apex with transversely oval swelling with radiating marginal macrotrichia.**

(2) *X.josifovi* M – ventral side with prominent ridge terminating in **small ventral tubercle** exceeding base of femur ventrad and only inconspicuous distad.

*(3) X.giganticus* F – ?

*(4) X.giganticus* (?) L 5 – ventral side with prominent strongly sclerotized **ridge-like projection** exceeding base of femur ventrad but not distad.

***Forefemur***

*(1) X.tomhenryi* F – strikingly thick and curved, arcuate, dorsal face convex, ventral face deeply and percurrently concave; groove delimited by two marginal rows of densely packed minute subglobular platelets of stronger sclerotization, anterior row of large granules more developed, on slightly elevated rim, granules in posterior row smaller, nearly subglobular proximally, becoming lens-like platelets distally, appearing in lower magnification as impression of two deeply brown to blackish lines. Surface of forefemoral concavity with countless lens-like platelets, heavily sclerotized, blackish, **the surface of the concavity densely pilose**. No other blackish platelets or granules present.

*(2) X.josifovi* M – distinctly curved, moderately C-shaped; ventral face concave, **with vestiture lacking**, parallel-sided and sharply delimited at anterior and posterior edges by row of macrotrichia and irregularly distributed black granules intermixed with row of conspicuous, high, non-setigerous conical tubercles.**Ventral concavity with numerous small, broad, transverse scale-like structures**. Blackish granules also on distal two thirds of lateral and dorsal faces.

*(3) X.giganticus* F – ?

*(4) X.giganticus* (?) L 5 – conspicuously curved, bearing numerous cuticular granules dorsally and ventrally.

***Foretibia***

*(1) X.tomhenryi* F – thick, flattish, rather uniformly broad, very moderately arcuate, ventral face deeply percurrently concave and **densely pilose**, concavity about as long as that of forefemur, margins of concavity delimited as in forefemur by subglobular platelets. Foretibia along ventral face (except proximal sixth) with two rows (posterior and anterior) of irregularly placed large, semi-globular to slightly conical, strongly sclerotized granules, appearing in lower magnification as impression of two deeply brown to blackish lines. No other blackish platelets or granules present.

*(2) X.josifovi* M –cylindrical, of uniform width, only dorsal outline slightly curved. **Entire ventral face moderately concave, vestiture lacking**, edges of tibial concavity less sharply delimited than those of femoral one. Anterior edge with 14, posterior edge with numerous conical tubercles of same shape as on femur. Anterior face with ca. 50 black granules, posterior face with several hundred. **Ventral concavity with numerous small, broad, transverse scale-like structures**.

*(3) X.giganticus* F – ?

*(4) X.giganticus* (?) L 5 –inner apical angle in form of pointed, strongly sclerotized projection bearing 3–5 straight, slender spines inserted below apex. Apex of central portion of inner surface of foretibia with field of short, stout setae.

***Apicitibial and tarsal armature of foreleg***

*(1) X.tomhenryi* F – tibial process **long and narrow, strikingly differentiated** from remainder of distal tibial edge, with **four slender and more dorsal spines, and one ventral thick, short, conical spine from more ventral tubercle.** Tarsal armature from four somewhat thicker setae (two anterior + two posterior = two dorsal + two ventral) distributed among normal macrotrichia, not longer than these, and recognizable only by presence of longitudinal grooving.

*(2) X.josifovi* M – tibial projection moderately large, rounded, with seven slender subapical spines, four in ventral row, three in dorsal row; tarsal armature from four spines, three of them very long and slender, one stout and conspicuously shorter.

*(3) X.giganticus* F – ?

*(4) X.giganticus* (?) L 5 – tibial projection acutangular but not markedly differentiated from distal edge of tibia; with 3–5 slender subapical spines.

***Pregenital abdomen, dorsum*** (a comparative study required)

*(1) X.tomhenryi* F – **complex lattice system (elevated rails and rungs)**

present.

*(2) X.josifovi* M – lattice absent.

*(3) X.giganticus* F – lattice absent.

*(4) X.giganticus* (?) L 5 – lattice absent.

## Discussion

*Nomenclatural and taxonomic notes.*Wygodzinsky and Schmidt (1991) did not explicitly exclude material of all the larvae of *X.giganticus* available to them (from Costa Rica, Panama, Colombia, and Guyana) from the type series of *X.giganticus*; these larvae must be considered paratypes, though their species identity is doubtful. The larva V from Colombia (Santa Marta: San Sebastian de Marago) used for completing the generic description and diagnosis of *Xenicocephalus* and illustrated by Wygodzinsky and Schmidt (1991: fig. 149) is also one of the paratypes of *X.giganticus*.

We cannot be certain which interspecific differences (Wygodzinsky and Schmidt 1991, Štys and Baňař 2008a, present paper) are real, and which should be interptreted as sexual dimorphism or intraspecific variation. However, the three adult specimens available are so different that their assignment to different species is beyond doubt. The architecture of the forelegs in *Xenicocephalus* and lattice system of abdominal carinae in *X.tomhenryi* are unique in the Enicocephalomorpha. We provisionally classify the genus as subfamily incertae sedis in the Enicocephalidae.

The distribution of *Xenicocephalus* can be characterized as “southern continental Central America and northern South America” (Wygodzinsky and Schmidt 1991), viz. Costa Rica; Panama: Canal Zone; Colombia: Cundinamarca, Magdalena (*X.giganticus*; Santa Marta; Ecuador: Pichincha (*X.tomhenryi*); Guyana; Suriname (*X.josifovi*). Only larvae of uncertain species identity are known from the areas with no species name provided. Species of *Xenicocephalus* species surely occur in the intervening and surrounding areas, and the number of species probably is higher than currently known.

Only scant information is available on the biology of *Xenicocephalus*. For example, pteygopolymorphism (adults of the three species are submacropterous to macropterous) and swarming are unknown. The male *X.josifovi* from Suriname was taken at light, whereas the other specimens were collected accidentally or in pitfall traps in lowland to montane forest (to 2600 m). The scattered data on collection dates do not provide useful information. However, the peculiar and, among Enicocephalomorpha (and perhaps all other Heteroptera), unique shapes of the forefemur and foretibia in *Xenicocephalus* suggest a specialized and unique mode of catching and handling a certain kind of prey. We predict that the curved, raptorial forefemora and foretibiae, both provided with an extensive and deep concave area on their ventral face, are suited for holding and possibly cracking strongly sclerotized, convex and rounded prey (as in similarly shaped beetles).

The larvae of species of *Xenicocephalus* (cf. Wygodzinsky and Schmidt 1991: fig. 149A) have the attributes of other larval Enicocephalidae. They are prothetelic, with distal parts of the forewing pads mutually contiguous and the mesoscutellar area distinctly circumscribed. The only available illustration of a larva of the genus also shows the small triangular apex of the mesoscutellum as sharply delimited on all sides, similarly structured, and probably sclerotized; this character has not been examined in other enicocephalid taxa.

## Supplementary Material

XML Treatment for Xenicocephalus

XML Treatment for Xenicocephalus tomhenryi
